# Leptomeningeal enhancement of myelin oligodendrocyte glycoprotein antibody-associated encephalitis: uncovering novel markers on contrast-enhanced fluid-attenuated inversion recovery images

**DOI:** 10.3389/fimmu.2023.1152235

**Published:** 2023-06-20

**Authors:** Li Li, Wen Liu, Qifang Cai, Yuqing Liu, Wenjing Hu, Zhichao Zuo, Qiuhong Ma, Siping He, Ke Jin

**Affiliations:** ^1^ Department of Radiology, Hunan Children’s Hospital, Changsha, Hunan, China; ^2^ Department of Radiology, The Third XiangYa Hospital, Central South University, Changsha, Hunan, China; ^3^ Department of Neurology, Hunan Children’s Hospital, Changsha, Hunan, China; ^4^ Department of Radiology, Xiangtan Central Hospital, Xiangtan, Hunan, China

**Keywords:** myelin oligodendrocyte glycoprotein, encephalitis, contrast enhancement, FLAIR, leptomeningeal

## Abstract

**Background:**

Myelin oligodendrocyte glycoprotein antibody disease (MOGAD) is a newly defined autoimmune inflammatory demyelinating central nervous system (CNS) disease characterized by antibodies against MOG. Leptomeningeal enhancement (LME) on contrast-enhanced fluid-attenuated inversion recovery (CE-FLAIR) images has been reported in patients with other diseases and interpreted as a biomarker of inflammation. This study retrospectively analyzed the prevalence and distribution of LME on CE-FLAIR images in children with MOG antibody-associated encephalitis (MOG-E). The corresponding magnetic resonance imaging (MRI) features and clinical manifestations are also presented.

**Methods:**

The brain MRI images (native and CE-FLAIR) and clinical manifestations of 78 children with MOG-E between January 2018 and December 2021 were analyzed. Secondary analyses evaluated the relationship between LME, clinical manifestations, and other MRI measures.

**Results:**

Forty-four children were included, and the median age at the first onset was 70.5 months. The prodromal symptoms were fever, headache, emesis, and blurred vision, which could be progressively accompanied by convulsions, decreased level of consciousness, and dyskinesia. MOG-E showed multiple and asymmetric lesions in the brain by MRI, with varying sizes and blurred edges. These lesions were hyperintense on the T2-weighted and FLAIR images and slightly hypointense or hypointense on the T1-weighted images. The most common sites involved were juxtacortical white matter (81.8%) and cortical gray matter (59.1%). Periventricular/juxtaventricular white matter lesions (18.2%) were relatively rare. On CE-FLAIR images, 24 (54.5%) children showed LME located on the cerebral surface. LME was an early feature of MOG-E (*P* = 0.002), and cases without LME were more likely to involve the brainstem (*P* = 0.041).

**Conclusion:**

LME on CE-FLAIR images may be a novel early marker among patients with MOG-E. The inclusion of CE-FLAIR images in MRI protocols for children with suspected MOG-E at an early stage may be useful for the diagnosis of this disease.

## 1 Introduction

Myelin oligodendrocyte glycoprotein antibody disease (MOGAD) is a newly defined autoimmune inflammatory demyelinating central nervous system (CNS) disease characterized by antibodies against the myelin oligodendrocyte glycoprotein (MOG) (1–3). MOGAD has a wide spectrum of clinical phenotypes, including MOG antibody-associated encephalitis (MOG-E), myelitis (MOG-M), and optic neuritis (MOG-ON), which may occur in isolation or in combination and vary with age (4–6). In older children and adults, MOG-ON is the most common phenotype, while MOG-E is the most common phenotype in children, particularly in younger children (7–9). Currently, the initial diagnosis of MOG-E is mainly based on clinical manifestations, magnetic resonance imaging (MRI), and antibody testing (10). However, the specific MRI features of MOG-E are still being explored.

More than 20 years ago, researchers demonstrated the use of the MOG protein in generating a mouse model of aseptic or autoimmune meningitis (11). MOG-E can present with an autoimmune or aseptic meningitis phenotype and can show leptomeningeal enhancement (LME) in MRI contrast-enhanced images (12–14). LME is an enhancement pattern that follows the leptomeningeal surface of the central nervous system and fills the subarachnoid spaces of sulci and cisterns, which reflects the disruption of the leptomeningeal blood barrier (15). The histopathological basis of these MRI findings is perivascular inflammation of the leptomeninges, involving B-cells, T-cells, and macrophages (16, 17). LME has been reported in patients with other diseases and interpreted as a biomarker of inflammation, including multiple sclerosis (MS), non-MS inflammatory neurologic diseases, non-inflammatory neurologic diseases, and human T-lymphotropic virus-infected and acquired immunodeficiency syndrome (18). Choosing a suitable MRI scanning sequence that can clearly show LME is important for the diagnosis of MOG-E. Currently, most studies on contrast-enhanced MRI for MOG-E still use traditional contrast-enhanced fat-suppressed T1-weighted images (CEFS-T1WI) (13, 19). However, other disease studies have shown that using contrast-enhanced fluid-attenuated inversion recovery (CE-FLAIR) images has more advantages than CEFS-T1WI in the identification of leptomeningeal lesions (18, 20, 21), suppressing the signal intensity from normal vascular structures on the cerebral surface and, consequently, making abnormal leptomeninges easier to visualize (21).

However, no reports have been published on contrast enhancement in the leptomeninges on the FLAIR images of children with MOG-E. Herein, we conducted a retrospective study and reported the prevalence and distribution of LME on CE-FLAIR images in children with MOG-E. Moreover, we described in detail the MRI features and clinical manifestations of MOG-E.

## 2 Methods

### 2.1 Participants

The study was approved by the institutional review board of Hunan Children’s Hospital. Informed consent was waived owing to the retrospective nature of this study, and all the procedures being performed were part of routine care.

**Figure 1:**
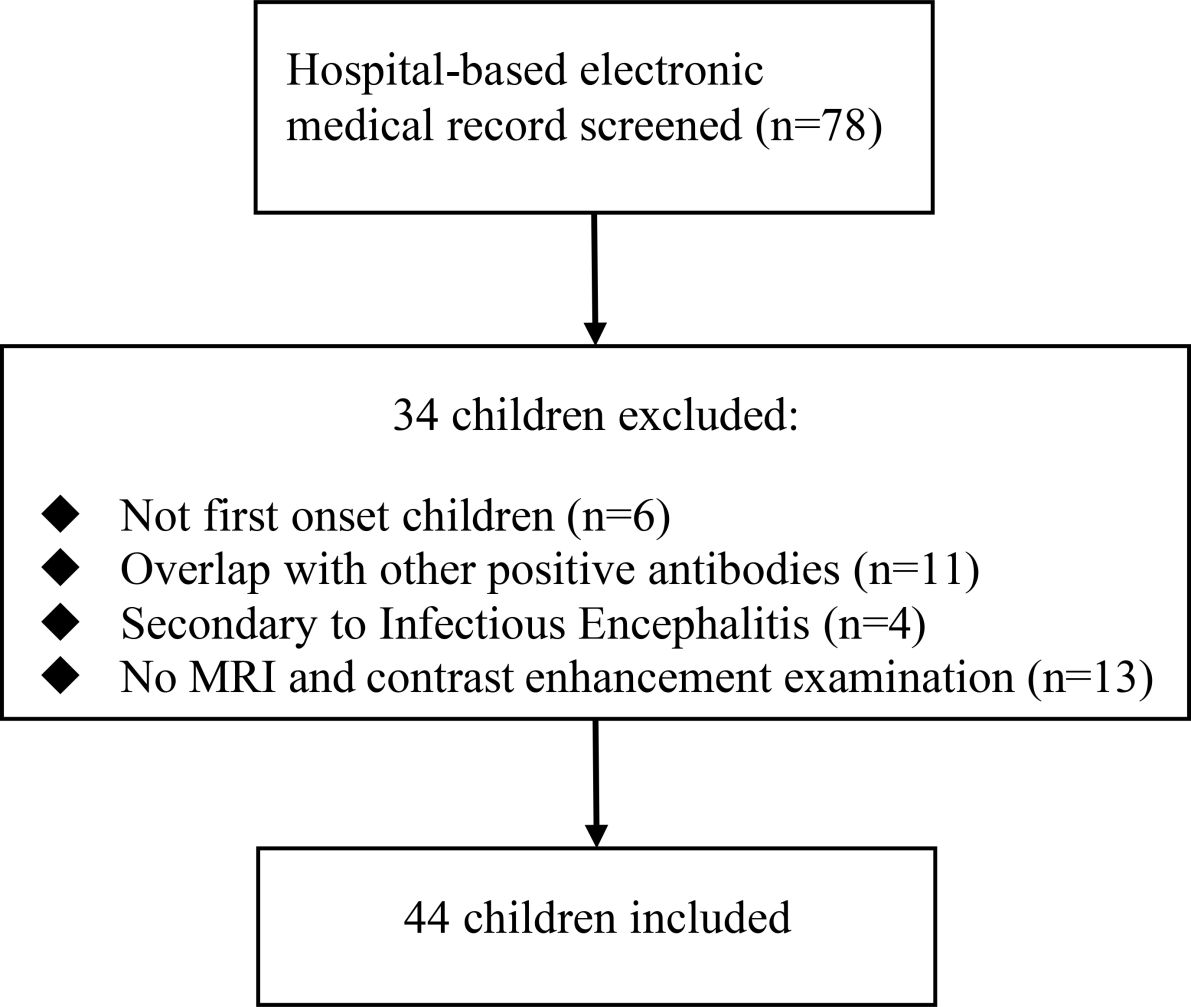
Overview of the patient selection process.

We retrospectively collected data from 78 children with MOGAD diagnosed at Hunan Children’s Hospital from January 2018 to December 2021. Patients who met the following criteria were included in the study: 1. Patients meeting the diagnostic criteria of MOGAD (10); 2. age ≤168 months (14 years old); and 3. medical records available. The exclusion criteria were a non-first-onset of MOGAD, coexistence of positive serum and/or CSF laboratory test for other auto-immune encephalitis, other diseases with brain, unavailability of MRI data, or limited image quality. Of the initial 78 participants, 34 were excluded (see **Figure 1** for the detailed selection process). For each patient, medical records, including demographics, clinical manifestations, and MRI findings, were reviewed.

### 2.2 Clinical data collection

The following data were collected: age at onset, sex, clinical symptoms, white blood cells (WBC) count, C-reactive protein (CRP), cerebrospinal fluid white blood cell (CSF WBC) count and CSF biochemistry. MRI results and the interval between symptom onset and MRI examination were also collected. The clinical symptoms at admission were divided into prodromal and progressive concomitant symptoms. The decreased level of consciousness is defined as a Glasgow Coma Scale (GCS) score of ≤14. Increased WBC in the serum was defined as a WBC >12 × 10^9^/L and divided into the mild (12–15 × 10^9^/L), moderate (15–20 × 10^9^/L), and severe (>20 × 10^9^/L) subgroups. CRP levels were deemed elevated if they were >8 mg/L. CSF WBC levels were deemed increased if they were >15 × 10^6^/L and divided into the mild (15–50 × 10^6^/L), moderate (50–100 × 10^6^/L), and severe (>100 × 10^6^/L) subgroups. CSF biochemistry included glucose (normal range: 2.8–4.2 mmol/L), chloride (normal range: 111–123 mmol/L), and protein (normal range: 0–0.5 g/L).

### 2.3 MOG-IgG assay

The serum and CSF samples were sent to two medical laboratories in China, the Kindstar Medical Laboratory and Guangzhou Medical Laboratory Center for antibody testing. Both laboratories utilized live cell-based assays (CBA) to analyze the antibody levels in the serum and CSF samples. Serum and CSF were initially diluted at ratios of 1:10 and 1:1, respectively.

### 2.4 MRI and image analysis

The children were randomly examined on two MRI devices (1.5-T and 3.0-T, Siemens, Munich, Germany). Children unable to cooperate with the examination were sedated using oral chloral hydrate anesthesia (0.5 mL/kg). The initial scanning sequence included axial T1-weighted images (T1WI), T2-weighted images (T2WI), FLAIR images, and sagittal T1WI. In addition, after manual injection of a single-dose contrast agent (0.1 mmol/kg, gadodiamide, GE Healthcare, Chicago, IL, USA), the scanning sequence included axial, coronal, and sagittal fat-suppressed T1WI, and axial FLAIR images. Details on MRI acquisition parameters are available in **Supplementary Table S1**.

Two radiologists independently reviewed all MRI scans in an unblinded fashion (L.L. and S.H., with 10 and 25 years of experience in neuroimaging, respectively). The images were reviewed with regard to lesion location, signal characteristics, and enhancement of brain parenchymal lesion and leptomeninges. Lesion location was classified as follows: within the cortical gray matter (GM), juxtacortical white matter (WM), deep WM, periventricular/juxtaventricular WM, basal ganglia, thalamus, hippocampus, brainstem, and cerebellum. LME and enhancement of brain lesions were evaluated on both CEFS-T1WI and CE-FLAIR images. In the case of disagreement, the radiologists reached a consensus through a discussion, with an inter-rater reliability of 95%.

### 2.5 Statistical analysis

We conducted an exploratory study of MOG-E. Data were tested for normal distribution, and normally distributed data are expressed as mean ± standard deviation (SD). In contrast, non-normally distributed data are expressed as median and inter-quartile range (IQR: Q1, Q3) after data testing for skewed distribution. Categorical variables are expressed as rates and composition ratios.

To test for normally and non-normally distributed data, we applied independent-sample t-tests and Mann-Whitney U tests. Enumeration data were compared with the chi-squared test or Fisher’s exact probability method. *P*-values < 0.05 were considered statistically significant.

## 3 Results

### 3.1 Demographics and clinical symptoms

**Figure 2:**
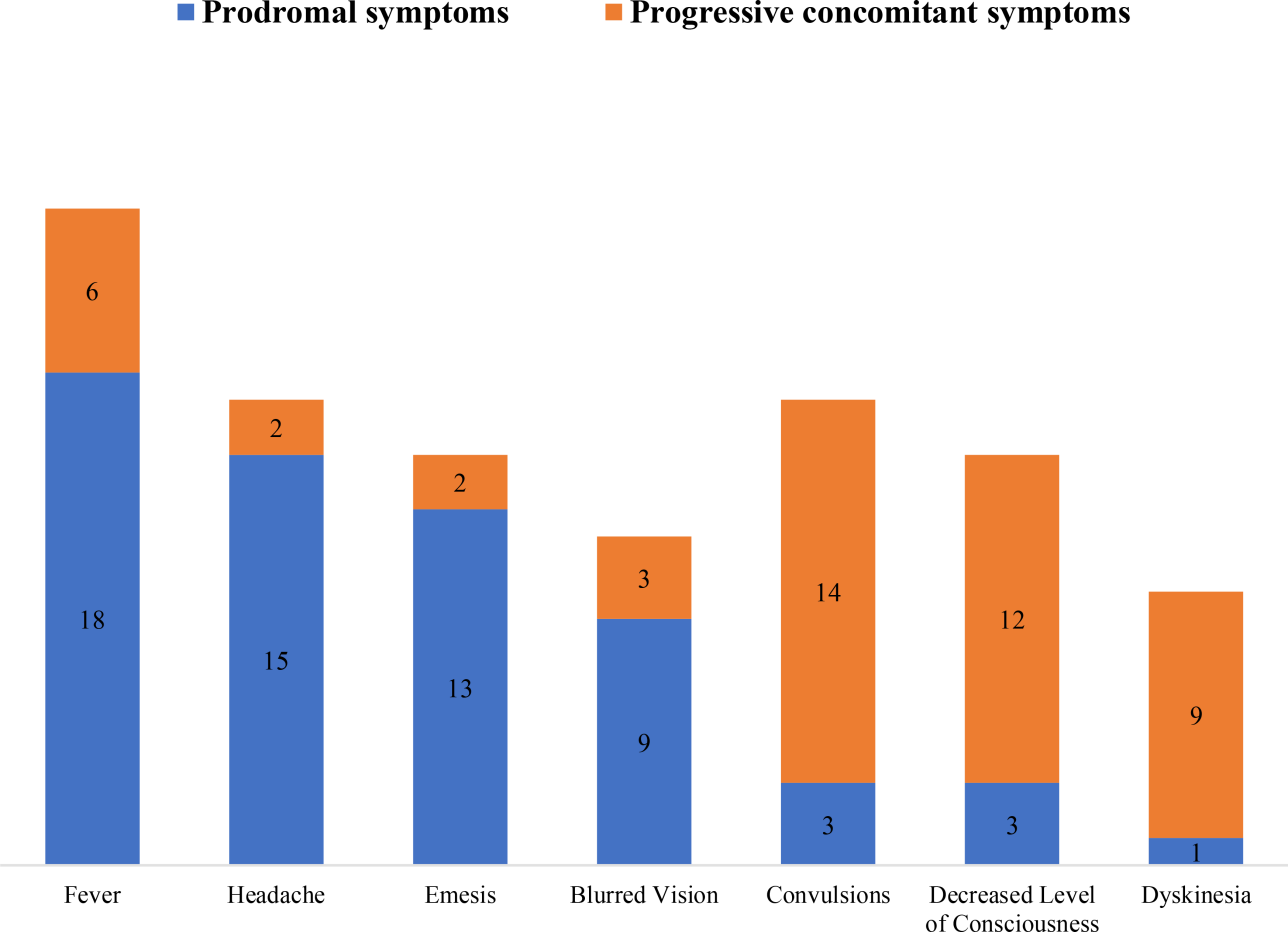
The prodromal symptoms and progressive concomitant symptoms of the study population.

**Table 1** summarizes the demographic and clinical data of 44 children with diagnosed MOG-E. The median age at the first-onset was 70.5 (range: 15–166) months, and the median interval between symptom onset and MRI examination was 10 days (IQR: 6, 20). The number of females was slightly higher than that of males (1.3:1). The prodromal symptoms in these children were fever, headache, emesis, and blurred vision, which could be progressively accompanied by convulsions; decreased level of consciousness, such as lethargy and coma; and dyskinesia, including unwillingness to walk and decreased muscle strength in the extremities (**Figure 2**).

**Table 1:** Characteristics of children with MOG-E with LME on CE-FLAIR images.

Demographics and Clinical Features	Total (n=44)	Contrast enhancement		*P*
		Present (n=24)	Absent (n=20)	
Age at onset, median (IQR) years, months	70.5 (47.5, 99.2)	60.5 (47.5, 106.2)	76 (51, 95.8)	0.981
Female, n (%)	25 (56.8)	14 (58.3)	11 (55)	1
Fever, n (%)	24 (54.5)	13 (54.2)	11 (55)	1
Headache, n (%)	17 (38.6)	9 (37.5)	8 (40)	1
Emesis, n (%)	15 (34.1)	11 (45.8)	4 (20)	0.139
Blurred Vision, n (%)	12 (27.3)	6 (25)	6 (30)	0.975
Convulsions, n (%)	17 (38.6)	11 (45.8)	6 (30)	0.445
Decreased Level of Consciousness, n (%)	15 (34.1)	8 (33.3)	7 (35)	1
Dyskinesia, n (%)	10 (22.7)	4 (16.7)	6 (30)	0.472
WBC (IQR×10^9^/L)	11.5(10.2, 13.4)	11.6 (10.9, 14.5)	10.8 (9.4, 12.5)	0.083
Normal	28 (63.6) 14	14 (58.3)	14 (70)	0.553
Mild, n (%)	9 (20.5)	6 (25)	3 (15)	
Moderate, n (%)	5 (11.4)	2 (8.3)	3 (15)	
Severe, n (%)	2 (4.5)	2 (8.3)	0 (0)	
CSF-WBC (IQR×10^6^/L)	45 (15, 74)	40 (12, 51.8)	53 (24.8, 81.5)	0.12
Normal	12 (27.3)	7 (29.2)	5 (25)	0.323
Mild, n (%)	16 (36.4)	11 (45.8)	5 (25)	
Moderate, n (%)	9 (20.5)	3 (12.5)	6 (30)	
Severe, n (%)	7 (15.9)	3 (12.5)	4 (20)	
Symptoms onset to MRI examination, median (IQR), days	10 (6, 20)	6.5 (4.8, 12)	18 (10, 30.2)	0.002
MRI areas affected				
Cortical GM, n (%)	26 (59.1)	16 (66.7)	10 (50)	0.417
Juxtacortical WM, n (%)	36 (81.8)	18 (75)	18 (90)	0.436
Deep WM, n (%)	11 (25)	3 (12.5)	8 (40)	0.08
Periventricular/Juxtaventricular WM, n (%)	8 (18.2)	3 (12.5)	5 (25)	0.436
Basal ganglia, n (%)	15 (34.1)	7 (29.2)	8 (40)	0.663
Thalamus, n (%)	17 (38.6)	7 (29.2)	10 (50)	0.27
Hippocampus, n (%)	10 (22.7)	4 (16.7)	6 (30)	0.472
Brainstem, n (%)	14 (31.8)	4 (16.7)	10 (50)	0.041
Cerebellum, n (%)	13 (29.5)	4 (16.7)	9 (45)	0.086
Brain parenchymal lesion enhancement, n (%)	11 (25)	6 (25)	5 (25)	1

IQR, interquartile range; WBC, white blood cells; CSF, cerebrospinal fluid; MRI, magnetic resonance imaging; GM, gray matter; and WM, white matter.

### 3.2 Laboratory data

The median serum WBC level was 11.5 (IQR: 10.2, 13.4) × 10^9^/L, and serum WBC levels increased in 16 (36.4%) children, of whom 9 (56.3%) had mildly increased levels. CRP was increased in 10 (22.7%) children. CSF was generally clear and transparent. However, the WBC in CSF was increased in 32 (72.7%) children, of whom 16 (50%) had mildly increased levels. The median CSF-WBC was 45 (IQR: 15, 74) × 10^6^/L, median CSF chloride was 120.6 (IQR: 115, 121) mmol/L, mean glucose was 3.1 ± 0.7 mmol/L, and median CSF protein was 0.3 (IQR: 0.2, 0.4) g/L, all of which were normal or only slightly abnormal.

### 3.3 MRI scans

**Figure 3:**
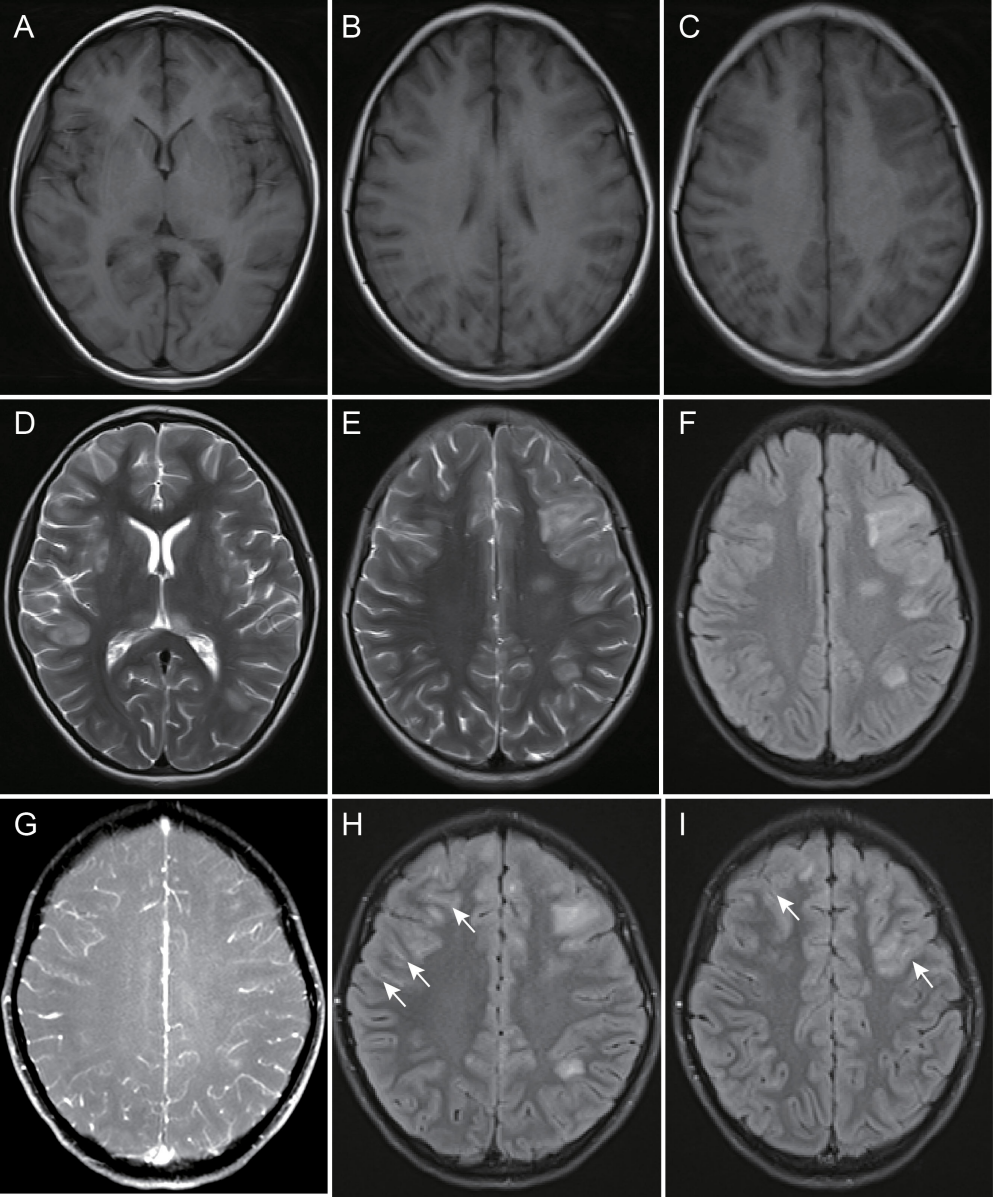
A 142-month-old female child with myelin oligodendrocyte glycoprotein antibody-associated encephalitis had a 10-day history of intermittent fever, which was accompanied by a decreased level of consciousness for 2 days. Magnetic resonance imaging showed multiple abnormal signals and blurred edges in the right basal ganglia, bilateral thalamus, left deep white matter, right temporal lobe, bilateral frontal parietal lobe cortex gray matter, and juxtacortical white matter, hypointensity on T1-weighted images **(A-C)**, and hyperintensity on T2-weighted and fluid-attenuated inversion recovery images **(D-F)**. There was no leptomeningeal enhancement on contrast-enhanced fat-suppressed T1-weighted images **(G)** and partial leptomeningeal enhancement in bilateral frontal lobe surface on contrast-enhanced fluid-attenuated inversion recovery images **(H, I)**, arrow).

In our included cohort, 16 children underwent brain scans using a 1.5-T MRI instrument, whereas 28 children underwent scanning using a 3.0-T MRI instrument. In general, MOG-E showed multiple and asymmetric lesions in the brain on MRI, with varying sizes and blurred edges. These lesions were hyperintense on the T2WI and FLAIR images and slightly hypointense or hypointense on the T1WI (**Figure 3**). The most common site involved was the juxtacortical WM (81.8%), followed by the GM (59.1%), thalamus (38.6%), basal ganglia (34.1%), brainstem (31.8%), cerebellum (29.5%), deep WM (25%), and hippocampus (22.7%). Lesions in the periventricular/juxtaventricular WM (18.2%) were relatively rare.

**Figure 4:**
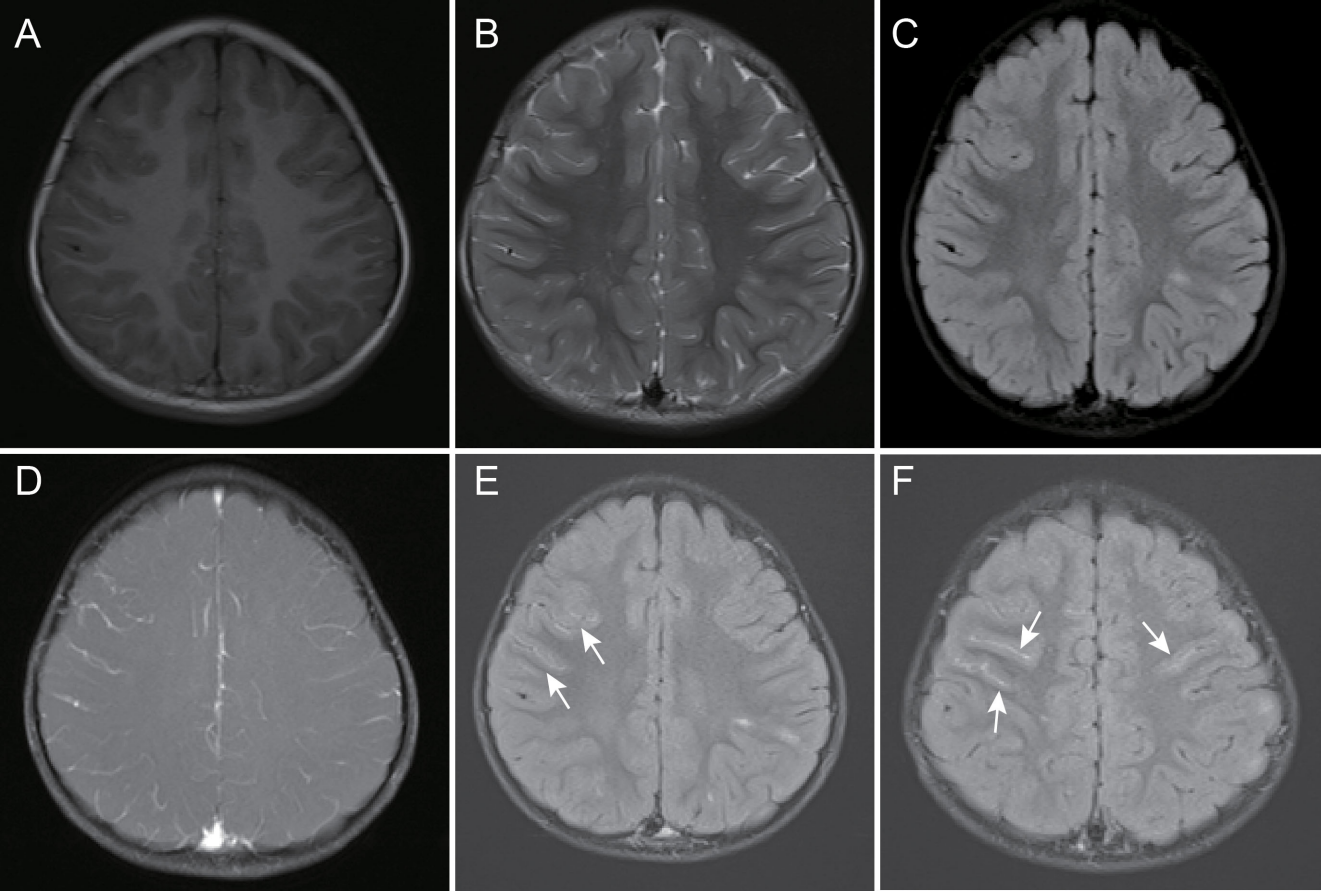
A 39-month-old female child with myelin oligodendrocyte glycoprotein antibody-associated encephalitis had a 2-day history of fever, headache, and emesis, accompanied by an episode of convulsion. Magnetic resonance imaging showed localized abnormal signal in the left parietal lobe juxtacortical white matter, and cortical gray matter, slight hypointensity on T1-weighted images **(A)**, hyperintensity on T2-weighted and fluid-attenuated inversion recovery images **(B, C)**, and thickening of blood vessels in the cerebral sulci of the right frontal lobe on contrast-enhanced fat-suppressed T1-weighted images and unobvious leptomeningeal enhancement **(D)** but partial bilateral leptomeningeal enhancement on contrast-enhanced fluid-attenuated inversion recovery images **(E, F)**, arrow).

After enhancement, 11 (25%) children showed lesion enhancement in the brain parenchyma, while 24 (54.5%) children showed LME on CE-FLAIR images, and in 4 (9.1%) children, this was the only pathological finding. Only 3 (6.8%) children showed LME on CEFS-T1WI images, which was not as obvious as that on CE-FLAIR images (**Figure 4**). The areas of LME were on the cerebral surface. There was no enhancement in the cistern, intraventricular meninges, and endocranium in any children. Two (4.5%) children had normal brain MRI but extracerebral lesions, which were isolated MOG-M and isolated MOG-ON.

### 3.4 Correlation between clinical symptoms and MRI findings

On CE-FLAIR images, there was a significant difference in the interval between the onset of symptoms to the MRI examination between the children with and without LME [6.5 days (IQR: 4.8, 12) vs. 18 days (IQR: 10, 30.2), *P* = 0.002]. This indicated that LME appeared in the early stage of the disease, and the probability of LME decreased as the disease progressed. In MOG-E, the brainstem was more easily affected in children without LME than in those with LME (*P* = 0.041). Other abovementioned clinical symptoms and MRI findings showed no statistically significant difference (**Table 1**).

## 4 Discussion

This study included a total of 44 children with MOGAD who underwent brain MRI enhancement. To the best of our knowledge, our report comprised the largest cohort used to study brain MRI enhancement in children with MOG-E.

In this study, we describe two novel findings regarding MOG-E. First, LME on MRI is a common manifestation in children with first-onset MOG-E. More than half of patients with MOG-E have LME on CE-FLAIR images. In some cases, this LME may be the only MRI finding. Second, LME may be the early MRI manifestation of MOG-E.

Previously, LME was reported in only a small number of case reports, rarely described in children with MOG-E (22–24), and considered to be an atypical MRI finding of MOG-E (25–27). In the current study, we found that LME in MOG-E is common on CE-FLAIR. However, the current use of CE-FLAIR is not common, with more traditional CEFS-T1WI, which has a lower detection rate, being used instead. Our study confirmed that LME on CEFS-T1WI is very rare. This could explain why LME is uncommon in patients with MOG-E in previous studies. Therefore, we suggest that the use of CE-FLAIR may help increase the sensitivity of detecting LME.

In addition, in our study cohort, children with a short interval from onset to MRI were more likely to show LME than those with a long interval. This is because, in the early stage of the disease, the MOG antibody in the peripheral serum passes through the blood-brain barrier and enters the central nervous system to bind to the nerve myelin sheath (3, 9). This process could lead to the destruction of the leptomeningeal vessel barrier on the brain surface. Therefore, MOG-E can manifest as autoimmune meningitis in the early stage, and LME can be seen on CE-FLAIR images.

The current study also summarized the clinical data and brain MRI involvement areas of 44 children with MOG-E. The median age of participants was 70.5 months, with no statistical significant sex difference, which is consistent with previous studies (12). Only two children presented with a normal brain, while the others had various brain abnormalities, confirming that encephalitis was predominant in children with MOGAD, especially in children aged less than nine years (1, 5). Consistent with previous reports, the prodromal symptoms of the first-onset disease were fever, headache, emesis, and blurred vision, which could be progressively accompanied by convulsions, decreased level of consciousness, and dyskinesia (28–30). Most of the infection indices in the blood of patients were normal or mildly abnormal. Although the WBC in the CSF were generally increased, they were only mildly increased in half of these instances, and the biochemistry of the CSF was normal or mildly abnormal, which was also consistent with the findings of other studies (1, 24, 31).

In our MRI study, we found that MOG-E showed multiple and asymmetric lesions in the brain, with varying sizes and blurred edges. The brain lesion was most likely to be located in the juxtacortical WM and cortical GM, followed by deep WM, brainstem, cerebellum, and deep WM (6, 32–34). Periventricular/juxtaventricular WM lesions were the least common, and only a few brain parenchymal lesions were enhanced, which was consistent with other studies (6, 31). Compared with children multiple sclerosis, MOG-E has an earlier age of onset and is more likely to have subcortical WM involvement. In particular, subcortical U-shaped fibers are easily involved, and the lesions are more widely distributed (12, 31).

This study had several limitations. First, because of the retrospective design of this study, the clinical symptoms were not detailed. Second, the study involved only a single center with a small sample size. Third, MRI studies were performed on scanners with different field strengths. To our knowledge, however, there is no discernible disparity between 1.5-T and 3.0-T MRI scanners for the routine clinical diagnosis of diseases, except for research applications, including functional MRI, spectroscopy, and automated lesion detection (35).Thus, future studies should comprise large-sample, multicenter, prospective MOG-E clinical trials using uniform MRI equipment and magnetic field strength protocols for further validation. Notwithstanding the study limitations, to the best of our knowledge, this is the first series investigating the additional value of LME on CE-FLAIR images during the first-onset of MOG-E in children.

In conclusion, our study support and extend previous observations in MOG-E. The prodromal clinical manifestations of MOG-E in children are fever, headache, emesis, or blurred vision, which could be progressively accompanied by neurological and/or mental disorders. In the MRI findings, MOG-E showed multiple and asymmetric lesions in the brain, with different sizes and blurred edges. The brain lesion was most likely to involve juxtacortical WM and cortical GM. LME on CE-FLAIR images may be a common early marker in MOG-E and possibly reflects a leptomeningeal inflammatory process, suggesting that it may be useful to include CE-FLAIR images in MRI protocols for children with suspected MOG-E. These findings will aid clinicians in detecting children likely to have MOG-E, as well as reduce excessive and futile tests and inefficient treatment practices.

## Data availability statement

The raw data supporting the conclusions of this article will be made available by the authors, without undue reservation.

## Ethics statement

The study was approved by the institutional review board of Hunan Children’s Hospital. Written informed consent to participate in this study was provided by the participants’ legal guardian/next of kin.

## Author contributions

LL and WL designed this study. LL analyzed the data and wrote the original draft of the manuscript. QC, WH, YL, and QM collected and interpreted the data. ZZ, SH, and KJ reviewed the manuscript. All authors contributed to the article and approved the final version.
