# Supplementary material for: Leptomeningeal enhancement of myelin oligodendrocyte glycoprotein antibody-associated encephalitis: uncovering novel markers on contrast-enhanced fluid-attenuated inversion recovery images
Source: Front Immunol. 2023 Jun 20;14:1152235. doi: 10.3389/fimmu.2023.1152235 (PMC10318903; doi:10.3389/fimmu.2023.1152235)
Supplement: Supplementary file 1 [file Table_1.docx]

**Supplementary Table S1.** Details on MRI acquisition parameters.

| **Model** | **MRI Sequence** | **TR, ms** | **TE, ms** | **Matrix** | **Field of View (FOV), mm** | **Slice Thickness, mm** |
| --- | --- | --- | --- | --- | --- | --- |
| **German, Siemens Skyra, 3.0T** | **Axial T1WI** | 1800 | 9 | 320×224 | 230×230 | 5 |
|  | **Axial T2WI** | 2200 | 100 | 384×268 | 230×230 | 5 |
|  | **Axial FLAIR** | 8000 | 100 | 320×224 | 230×230 | 5 |
|  | **Sagittal T1WI** | 200 | 2.5 | 320×256 | 250×250 | 4 |
|  | **Axial CEFS-TIWI** | 450 | 2.5 | 320×256 | 220×220 | 5 |
|  | **Sagittal CEFS-TIWI** | 450 | 2.5 | 288×216 | 220×220 | 4 |
|  | **Coronal CEFS-TIWI** | 450 | 2.5 | 320×256 | 220×220 | 5 |
|  | **Axial CE-FLAIR** | 9000 | 100 | 320×224 | 230×230 | 5 |
| **German, Siemens Avanto, 1.5T** | **Axial T1WI** | 550 | 15 | 256×230 | 230×230 | 5 |
|  | **Axial T2WI** | 4500 | 100 | 256×230 | 230×230 | 5 |
|  | **Axial FLAIR** | 9000 | 96 | 256×198 | 230×230 | 5 |
|  | **Sagittal T1WI** | 500 | 10 | 256×230 | 250×250 | 4 |
|  | **Axial CEFS-TIWI** | 250 | 5 | 256×218 | 230×230 | 5 |
|  | **Sagittal CEFS-TIWI** | 250 | 5 | 256×230 | 200×200 | 5 |
|  | **Coronal CEFS-TIWI** | 250 | 5 | 256×218 | 200×200 | 5 |
|  | **Axial CE-FLAIR** | 9000 | 100 | 256×212 | 230×230 | 4 |

Abbreviations: ITR, Repetition Time; TE, Echo Time**;** FOV, Field of View; CEFS-T1WI, contrast-enhancement fat-suppressed T1-weighted images; CE-FLAIR, contrast-enhancement fluid-attenuated inversion recovery; T1WI, T1-weighted images; T2WI, T2-weighted images.
